# Crystal structure of Onconase at 1.1 Å resolution – insights into substrate binding and collective motion

**DOI:** 10.1111/j.1742-4658.2011.08320.x

**Published:** 2011-11

**Authors:** Daniel E Holloway, Umesh P Singh, Kuslima Shogen, K Ravi Acharya

**Affiliations:** 1 Department of Biology and Biochemistry, University of BathUK; 2 Cedar Grove LaneSomerset, NJ, USA

**Keywords:** atomic displacement parameters, elastic network model, ribonuclease, X-ray crystallography

## Abstract

Onconase® (ONC) is an amphibian member of the pancreatic ribonuclease superfamily that is selectively toxic to tumor cells. It is a much less efficient enzyme than the archetypal ribonuclease A and, in an attempt to gain further insight, we report the first atomic resolution crystal structure of ONC, determined in complex with sulfate ions at 100 K. The electron density map is of a quality sufficient to reveal significant nonplanarity in several peptide bonds. The majority of active site residues are very well defined, with the exceptions being Lys31 from the catalytic triad and Lys33 from the B_1_ subsite, which are relatively mobile but rigidify upon nucleotide binding. Cryocooling causes a compaction of the unit cell and the protein contained within. This is principally the result of an inward movement of one of the lobes of the enzyme (lobe 2), which also narrows the active site cleft. Binding a nucleotide in place of sulfate is associated with an approximately perpendicular movement of lobe 2 and has little further effect on the cleft width. Aspects of this deformation are present in the principal axes of anisotropy extracted from C^α^ atomic displacement parameters, indicating its intrinsic nature. The three lowest-frequency modes of ONC motion predicted by an anisotropic network model are compaction/expansion variations in which lobe 2 is the prime mover. Two of these have high similarity to the cryocooling response and imply that the essential ‘breathing’ motion of ribonuclease A is conserved in ONC. Instead, shifts in conformational equilibria may contribute to the reduced ribonucleolytic activity of ONC.

**Database:**

Structural data have been submitted to the Protein Data Bank under accession number 3SNF.

## Introduction

Onconase® (ONC, Alfacell Corporation, Somerset, NJ, USA) is a ribonuclease with anti-tumor activity isolated from oocytes of the Northern leopard frog, *Rana pipiens* [1]. It is selectively toxic toward numerous cancer cell types [2] and represents a promising anti-cancer drug, having undergone a confirmatory Phase IIIb clinical trial for the treatment of nonresectable malignant mesothelioma [3].

**Figure 1:**
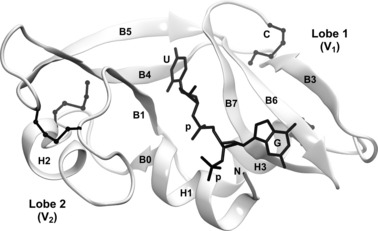
ONC topology. Schematic representation of ONC bound to a dinucleotide substrate analogue as derived from the crystal structure of the ONC·d(AUGA) complex (PDB entry 2I5S) [10]. Helices H1–H3 and β-strands B0–B7 are labeled, and are numbered for consistency with the structural elements of RNase A (a short loop is found in place of β-strand B2 of RNase A). The foundations of lobes 1 and 2 are β-sheets V_1_ (B3, B6 and B7 comprising residues 55–58, 86–91 and 96–101, respectively) and V_2_ (B0, B1, B4 and B5 comprising residues 11–12, 33–38, 63–70 and 77–84, respectively) [8,9]. The active site lies in a cleft between the lobes, here occupied by a d(UpGp) moiety shown in stick representation. The four disulfide bonds are shown in ball-and-stick representation (the one connecting H3 and B6 is obscured).

ONC is a member of the pancreatic ribonuclease superfamily whose archetypal member, ribonuclease A (RNase A; EC 3.1.27.5), holds a central position in the study of protein chemistry, enzymology and molecular evolution [4–6]. Although ONC has only 30% sequence identity with RNase A, contains 20 fewer residues (104 versus 124) and possesses an alternative disulfide bonding arrangement, it has a highly similar α/β fold [7]. The structure is bi-lobed, with the lobes founded on β-sheets designated V_1_ and V_2_, respectively [8,9] (Fig. 1). Canonically, the active site lies in the cleft between the two lobes [10].

The ribonucleolytic activity of ONC is essential to its cytotoxicity, and the target of this activity is intracellular RNA [11]. In common with RNase A, RNA cleavage is endonucleolytic, occuring specifically on the 3′-phosphate side of pyrimidine residues [12,13]. However, whereas RNase A is an efficient enzyme exhibiting *k*_cat_/*K*_m_ values in excess of 10^7^ m^−1^·s^−1^ with optimized oligonucleotide substrates [14], ONC is much less so under equivalent conditions (by ∼ 10^3^-fold) [13]. Research to date suggests that the physical basis for this derives not only from localized structural features, but also from differences in the collective dynamics of the two enzymes. The body of work on RNase A is extensive [6] and has shown that RNase A interacts with the central dinucleotide of the substrate via multiple interactions spread across a tightly-fitting binding site [15,16]. Cleavage occurs across the P–O^5′^ bond by a two-step general acid–base mechanism involving a catalytic triad in which two histidine residues (His12 and His119) orchestrate the movement of protons [17], and a lysine residue (Lys41) stabilizes the transition state via donation of a hydrogen bond to a nonbridging phosphoryl oxygen [18]. In addition, RNase A is known to interconvert between open and closed forms [8,19–24]. The motion is subtle but appears to be coupled to turnover [25], with an open conformation associated with substrate encounter and a closed one with catalysis [24,26]. Data relating to ONC are less abundant, although the enzyme is known to differ from RNase A in several ways: it binds the substrate less tightly (by ∼ 10^2^-fold so) [13] in a groove that is significantly wider [7,10]. Lys31 (similar to Lys41, its counterpart in RNase A) is critical for catalysis [13] but most likely requires a substantial (and thus far unobserved) change in enzyme conformation to participate [7,10]. An additional lysine residue, Lys9, also plays a major role in catalysis [13]. Collective motion comparable to that of RNase A has not been detected, either by essential dynamics simulations of the wild-type enzyme [9] or by application of dynamic NMR techniques to the <Q1S mutant [27].

Our understanding of RNase A and other ribonucleases has benefited greatly from their characterization at atomic or near-atomic resolution [22,28–30]. By contrast, all ONC structure–function studies to date have used a significantly lower, 1.7 Å resolution structure as a reference point [7]. Redressing this, we have determined the X-ray crystal structure of ONC at atomic resolution. It provides a more precise description of the active site region, its geometry and atomic fluctuations, and allows a refined interpretation of the changes that occur upon nucleotide binding [10] and mutation [9]. Furthermore, via the combined evaluation of peptide backbone alignments, anisotropic atomic displacement parameters (ADPs) and normal mode analysis using an elastic network model, we identify collective motion in the ONC structure that may have significance for turnover. This provides valuable insight into the interplay between molecular structure and collective dynamics during the divergent evolution of the pancreatic ribonuclease superfamily.

## Results

### Structural overview

The atomic resolution structure of ONC·SO_4_ was determined from an orthorhombic crystal at 100 K (Tables 1 and 2). The previously reported ONC·SO_4_ structure, determined at room temperature in the same crystal form to 1.7 Å resolution [Protein Data Bank (PDB) entry 1ONC] [7] is hereafter referred to as rtONC·SO_4_. The present structure was compared in detail with rtONC·SO_4_ and also with three significant low-temperature ONC crystal structures: one in the same crystal form [M23L-ONC·SO_4_ at 1.51 Å resolution (PDB entry 1YV4)] [9] and two in different crystal forms [the ONC·d(AUGA) complex at 1.9 Å resolution (PDB entry 2I5S) and the T89N/E91A-ONC·5′-AMP complex at 1.65 Å resolution (PDB entry 2GMK)] [10]. Sigma A-weighted electron density maps (as supplied by the Uppsala Electron-Density Server) [31], as well as the coordinates of all structures, were examined.

**Table 1:** Crystallographic statistics.

Characteristic	Value
X-ray wavelength (Å)	0.978
Resolution range (Å)	32.0–1.1
Space group	P2_1_2_1_2_1_
Unit cell dimensions (Å)	*a* = 32.23, *b* = 38.16, *c* = 68.92
Number of reflections^a^	
Measured	110 620
Unique	30 093
*R*_symm_^b^	0.059 (0.330)^c^
*I*/σ(*I*)	22.4 (2.8)^c^
Completeness (%)^b^	85.1 (68.0)^c^
Wilson *B*-factor	9.1

a Data cut-off: *I* > −3σ(*I*).

b *R*_symm_ = Σ_h_Σ_i_[|*I*_i_(*h*) − 〈*I*(*h*)〉|/Σ_h_Σ_i_*I*_i_(*h*)], where *I*_i_ is the *i *th measurement and 〈*I*(*h*)〉 is the weighted mean of all measurements of *I*(*h*).

c Values in parentheses refer to the outermost shell (1.12–1.10 Å).

**Table 2:** Structure refinement statistics.

Characteristic	Value
Number of non-hydrogen atoms^a^	
Protein	842
Anions	29
Water	149
Total	1020
Number of observations	29 163
Number of parameters	9193
Number of restraints	2687
(Observations + restraints)/parameters	3.5
*R*_work_^b^	0.161
*R*_free_^c^	0.183
Mean anisotropy^d^	0.57 ± 0.14
Deviation from ideality (rmsd)	
Bond lengths (Å)	0.016
Bond angles (°)	1.77
Mean isotropic equivalent *B*-factor (Å^2^)	
Protein atoms	12.1
Anions	17.0
Water molecules	20.9
All atoms	13.4

a Atoms in alternate conformations are included.

b *R*_work_ = Σ_h_ |*F*_o_−*F*_c_|/Σ_h_*F*_o_, where *F*_o_ and *F*_c_ are the observed and calculated structure factor amplitudes of reflection *h*, respectively.

c *R*_free_ is equal to *R*_work_ for a randomly selected 5% of reflections not used in the refinement [61].

d Calculated with parvati [69].

The final atomic model fits the experimental data well (Table 2). The entire main chain is well defined in the 2*F*_o_−*F*_c_ electron density map and, of the 95 eligible residues, 83 lie in the most favored regions of the Ramachandran plot, with the remaining 12 in the additional allowed regions. This is similar to the previous rtONC·SO_4_, M23L-ONC·SO_4_ and ONC·d(AUGA) structures but is closer to ideality than the T89N/E91A-ONC·5′-AMP structure in which 17 residues lie in the additional allowed regions.

The enhanced resolution of the data permitted the relaxation of stereochemical restraints during crystallographic refinement, providing extra detail throughout the structure. A clear illustration of this is provided by a segment involving the start of helix H3; here, the main chain adopts a strained conformation in which the peptide bonds of Ser39, Arg40 and Pro41 exhibit significant deviations from planarity (ω dihedral angles = 160.0, 192.1 and 193.5°, respectively). The conformations of the side chains of Arg40 and Glu42 are well defined, with the guanidino group of Arg40 coordinating a sulfate ion. These features are not apparent in the rtONC·SO_4_ structure (which was refined with a relatively narrow spread of ω) and contribute to a significant shift in the main chain in this region, notably a > 1 Å change in the position of the C^α^ atom of Arg40.

The data also enabled the resolution of discrete alternate conformations for the side chains of six residues (Asp18, Ile51, Ser54, Thr60, Thr83 and Asn84) to the extent that their 2*F*_o_−*F*_c_ electron density is continuous when contoured at up to 1.4 σ. This contrasts with the rtONC·SO_4_, M23L-ONC·SO_4_ and ONC·d(AUGA) structures, which specify no alternate conformations, and with the T89N/E91A-ONC·5′-AMP structure, where, of the non-mutated residues modeled in alternate conformations (Asn26, Lys33, Arg40, Glu42 and Lys80), only Asn26 has continuous 2*F*_o_−*F*_c_ electron density, even when the contouring level is reduced to 1.0 σ.

### Active site residues

The structure provides a refined view of the active site as it occurs in the absence of nucleotide bases or sugars. In accordance with the convention devised for RNase A [32], it can be viewed as a series of subsites that bind the successive base and phosphoryl groups of the RNA substrate. The scissile phospodiester linkage is bound at the P_1_ subsite, whereas the bases located immediately upstream and downstream (5′→3′) are bound at the B_1_ and B_2_ subsites, respectively.

**Figure 2:**
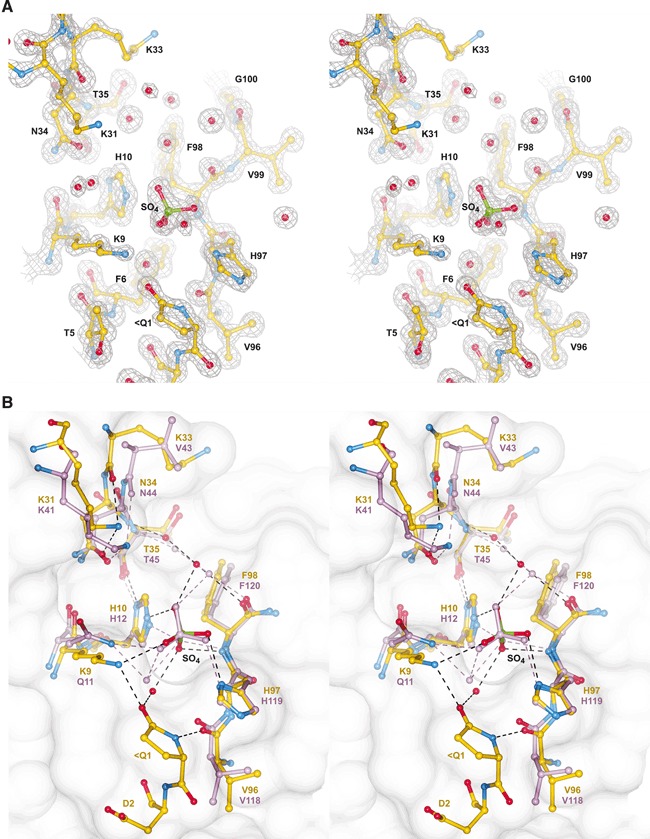
Active site. (A) Electron density map in the region of the P_1_ and B_1_ subsites. Shown in stereo are a wireframe representation of the final 2*F*_o_−*F*_c_ electron density map (contoured at 1.6 σ) and ball-and-stick representations of the protein and the bound sulfate ion. Carbon, nitrogen, oxygen and sulfur atoms are colored gold, blue, red and green, respectively. Spheres denote water molecules and ‘<Q1’ is the N-terminal pyroglutamate residue. The majority of residues are very well defined, with the exceptions being Lys31 whose side-shain is disordered beyond C^δ^, and Lys33, whose side-chain is poorly defined (occupancy = 0.61) beyond C^β^. (B) Comparison of sulfate binding by ONC and RNase A. The ONC·SO_4_ and RNase A·SO_4_ (PDB entry 1KF2) [22] structures were superposed on the basis of the C^α^ positions of selected nucleotide-binding residues (from ONC, H10, T35 and H97; from RNase A, H12, T45 and H119). Shown in stereo are ball-and-stick representations of each complex along with a surface representation of ONC. The color scheme for ONC·SO_4_ is the same as for (A), whereas that for RNase A·SO_4_ differs in that its carbon atoms and sulfate ion are colored pink. Potential hydrogen-bonds are shown as dashed lines (in black for ONC·SO_4_, in pink for RNase A·SO_4_). For those RNase A residues that are observed in dual conformations, the one that most closely matches the conformation of the corresponding ONC residue is shown (conformation *A* of Q11 and V43; conformation *B* of K41, H119 and the sulfate ion).

The majority of active site residues are very well defined in the electron density map (Fig. 2A) and occupy positions similar to those in the rtONC·SO_4_ structure. Hence, at the P_1_ subsite, residues Lys9, His10, His97 (the latter in the inactive, *B* conformation) and Phe98 (the counterparts of RNase A residues Gln11, His12, His119 and Phe120, respectively) coordinate a sulfate ion (Fig. 2B). The N-terminal pyroglutamate residue (Pca1), which serves to align Lys9 for catalysis [13], is now refined in a puckered conformation rather than the planar one described by the rtONC·SO_4_ coordinates. As noted previously [7], Lys31 (the counterpart of RNase A Lys41) lies somewhat further afield. The refinement directs its side chain into a more extended conformation than that reported for rtONC·SO_4_, placing the N^ζ^ atom 5.22 Å from the nearest sulfate O atom (cf. 5.37 Å in rtONC·SO_4_). This leaves it within hydrogen-bonding range of the O atom of Lys33 (2.92 Å distant) and the O^δ1^ atom of Asn34 (3.03 Å distant), although it should be noted that electron density for Lys31 is lacking beyond C^δ^, indicating that the side chain of this residue has significant mobility and most likely makes no firm hydrogen bonds in the presence of sulfate. Reinspection of respective electron density maps indicates that Lys31 is disordered to a similar extent in rtONC·SO_4_ but adopts a well-defined, extended conformation in the ONC·d(AUGA) complex.

There is little positional change among the majority of the residues that line the pyrimidine-binding pocket (B_1_ subsite). In addition to His10 and Phe98 noted above, this includes Asn34, Thr35, Val99, Gly100 and Val101. The exception is Lys33 (the counterpart of RNase A Val43) whose side chain stacks against the uracil moiety in the ONC·d(AUGA) structure [10]. The side chain of this residue is partially disordered with weak electron density beyond C^β^ (Fig. 2A), and the refinement suggests that the major side chain rotamer intrudes into the space occupied by the bound pyrimidine. In comparison, the rtONC·SO_4_ structure presents the side chain in a more extended conformation and the disorder, although detectable, is not as pronounced. Upon binding the uracil moiety of d(AUGA), the side chain adopts a well-defined, extended conformation.

At the B_2_ subsite (a shallow depression lined by Thr89, Glu91, Val96 and His97), there is little to distinguish the present and the rtONC·SO_4_ structures.

### Main-chain dynamics

**Figure 3:**
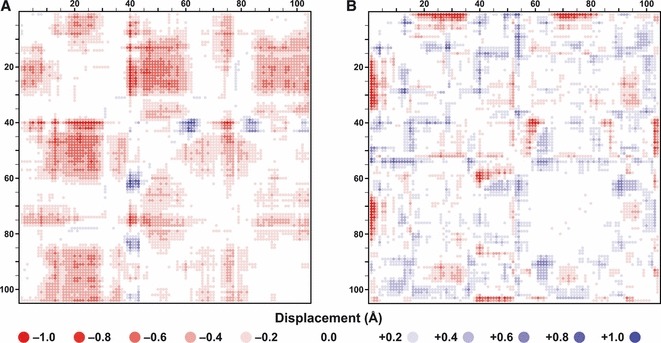
Difference distance analysis of changes in main-chain conformation. Heat maps for difference distance matrices obtained after (A) cryocooling (rtONC·SO_4_ → ONC·SO_4_) and (B) exchange of a sulfate for a dinucleotide at the active site [ONC·SO_4_ → ONC·d(AUGA)]. Negative values (red dots) signify C^α^ pairs that move closer together, whereas positive values (blue dots) signify the converse.

**Figure 4:**
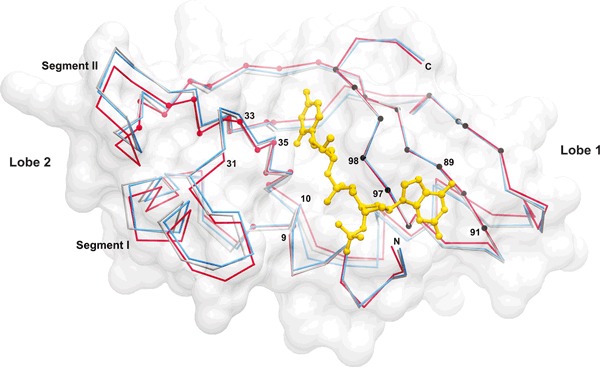
Main-chain movement in response to cryocooling and dinucleotide binding. C^α^ traces of ONC extracted from the crystal structures of rtONC·SO_4_ (PDB entry 1ONC) [7] (gray), ONC·SO_4_ (present study) (blue) and ONC·d(AUGA) (PDB entry 2I5S) [10] (red), superposed on the basis of the V_1_β-sheet residues (black studs). V_2_β-sheet residues are highlighted with red studs, whereas two relatively mobile segments (I and II) of lobe 2 are labeled, as are the positions of key substrate-binding residues. To highlight the active site cleft, the protein surface and the active site ligand, d(UpGp) (ball-and-stick) from the ONC·d(AUGA) structure are superimposed. The orientation is identical to that of Fig. 1.

**Figure 5:**
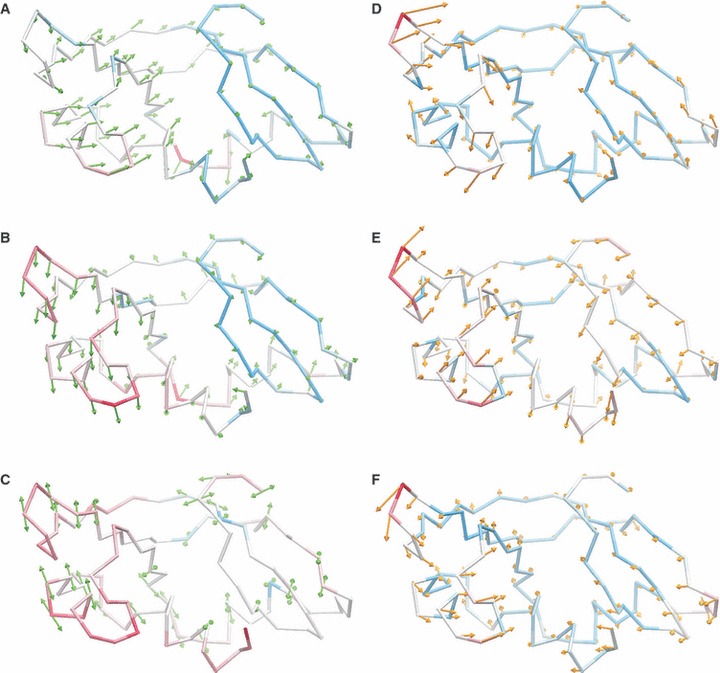
Main-chain dynamics from experiment and theory. In each panel, a C^α^ trace of ONC is shown, oriented as in Figs 1 and 4 and colored by an index of mobility (blue represents low; white, intermediate; red, high mobility). (A, B) Information from crystal structure alignment. The rtONC·SO_4_, ONC·SO_4_ and ONC·d(AUGA) crystal structures were superposed on the basis of the V_1_β-sheet residues, as in Fig. 4. (A) Showing rtONC·SO_4_, colored according to the C^α^ displacements resulting from cooling to 100 K (range = 0.02–1.14 Å). (B) Showing ONC·SO_4_, colored according to the C^α^ displacements resulting from the exchange of sulfate for d(AUGA) and crystallization in an alternative space group (range = 0.04–1.70 Å). Arrows represent the C^α^ deformation vectors (up-scaled by an arbitrary factor for clarity). (C) Information from the atomic displacement parameters of the ONC·SO_4_ crystal structure. The ONC·SO_4_ trace is colored according to the *B*_eq_ values of the C^α^ atoms (range = 7.6–15.8 Å^2^). Arrows depict the principal axes of positional fluctuation for those C^α^ atoms whose anisotropy exceeds the mean by half the standard deviation or more (i.e. *A* < 0.5565), scaled in proportion to the respective eigenvalue. (D–F) The three lowest frequency nontrivial modes of motion (numbers 7–9, respectively) predicted by ANM. Traces of ONC·SO_4_ are colored according to the relative mobility at each position; arrows show the direction and magnitude of motion during the compaction phase of each mode (arbitrary scaling).

Comparison with the rtONC·SO_4_ data reveals that the low temperature used during X-ray diffraction brings about a 7.7% compaction in unit cell volume, with the largest reduction (5.8%) affecting the *b* axis. This is coincident with a compaction of the protein structure, as judged by a 2.0% reduction in solvent-excluded volume (from 13685 to 13409 Å^3^) and a 1.0% reduction in radius of gyration (from 13.736 to 13.603 Å). After global alignment, the C^α^ traces of the two structures differ by only 0.35 Å (rmsd). However, difference distance analysis [33] of the two structures reveals a widespread reduction in inter-C^α^ distances (Fig. 3A). Furthermore, it indicates that a significant contribution to the compaction is made by the movement of lobes 1 and 2 towards one another. For example, each of the strands of the V_1_β-sheet (B3, B6 and B7, comprising residues 55–58, 86–91 and 96–101, respectively) moves closer to each of those of V_2_ (B0, B1, B4 and B5, comprising residues 11–12, 33–38, 63–70 and 77–84, respectively). The largest attractive movements are measured for the loops and helices that decorate the periphery of the lobes, in particular the segments in lobe 2 that connect B0 to B1 (residues 13–32, incorporating helix H2) and B4 to B5 (residues 71–75), and the one in lobe 1 that connects B1 to B3 (residues 39–54, incorporating helix H3). The conformational change can be visualized most effectively by aligning the structures on the basis of the V_1_β-sheet residues alone (Figs 4 and 5A). This presents an inward movement of the V_2_β-sheet and the attached B0–B1 segment that encompasses H2 and its flanking loops (‘segment I’). Some of the main chain components of the active site cleft are affected. For example, the distance between the main-chain N atoms of Thr35 and Phe98 (which are located on opposite sides of the cleft and have been used to gauge its width) [8,9] reduces from 9.17 to 8.86 Å.

Differences in main chain conformation are also detectable when the structure is compared with that of the low-temperature ONC·d(AUGA) complex. Overall, the C^α^ traces of these two structures differ by only 0.38 Å (rmsd) but, again, there are marked deviations at the periphery of lobe 2. After a V_1_ alignment, a concerted motion of two of the lobe 2 segments is evident (Figs 4 and 5B). One of these is segment I and the other comprises the tips of B4 and B5 and the intervening loop (‘segment II’); the two segments are bridged by a disulfide bond (Fig. 1). The segments move approximately parallel to the active site cleft in the direction of the N-terminus (effectively down the nucleotide backbone) but do not bring about further compaction of the structure. Indeed, the protein component of the complex has a solvent-excluded volume (13430 Å^3^) and radius of gyration (13.636 Å) that are 0.2% larger than those of the present structure, and the difference distance plot indicates an approximately even balance of positive and negative C^α^ displacements (Fig. 3B). Several residues at the active site cleft are affected, notably Lys31, whose C^α^ atom moves away from the base at B_1_ and toward the phosphate at P_1_, as well as Lys9 and Thr35, whose C^α^ atoms retreat by a smaller margin from the substrate analog. The movement reduces the Thr35 N···N Phe98 distance only slightly to 8.76 Å.

Elements of the aformentioned main-chain dynamics are discernible among the ADPs of the present structure. The isotropic equivalent *B*-factors (*B*_eq_) of the C^α^ atoms are in the range 7.6–15.8 Å^2^, with a mean of 10.3 ± 1.6 Å^2^. The majority of the largest *B*_eq_ values are located in lobe 2 with noticeable concentrations in segments I and II (Fig. 5C), confirming that this region is a relatively mobile part of the structure. The anisotropy (*A*) of the C^α^ atoms is in the range 0.278–0.856, with a mean of 0.610 ± 0.107. Of those C^α^ atoms with relatively high anisotropy (*A* below the mean by half the standard deviation or more), a high proportion is located in segments I and II (Fig. 5C). Here, the principal axes of the C^α^ fluctuations are an excellent match for the axes of the displacements deduced from structural alignment with the ONC·d(AUGA) complex (Fig. 5B), indicating that, after the binding of sulfate, these segments have an intrinsic propensity to undergo this motion.

Intrinsic collective motion was further investigated using the anisotropic network model (ANM) with normal mode analysis. The three lowest-frequency nontrivial modes (numbers 7–9) are found to describe variations on the compaction/expansion (‘breathing’) theme (Fig. 5D–F). In each case, there is a counter-motion of the two lobes, with lobe 2 undergoing the larger movement. Modes 7 and 9 also feature a propeller-like twisting of lobe 2, such that segments I and II move in opposite directions relative to the active site cleft. When viewed during the equivalent phase of motion (e.g. compaction), it is evident that the twist directions in these two modes are opposed. Mode 8, on the other hand, describes a pincer-like counter-motion of the two lobes in which segments I and II move in unison. This is reminiscent of the breathing motion identified in RNase A via crystallographic analyses [8,20,23,24] and molecular dynamics simulations [34–36].

The compaction phases of modes 8 and 9 have much in common with the response of crystalline ONC·SO_4_ to cryocooling (Fig. 5A), although they both overestimate the movement of segment II. A plausible explanation for this lies in the intimate involvement of this segment in crystal packing interactions that constrain its movement. The additional movement of lobe 2 that accompanies nucleotide binding (Fig. 5B) may have its origins in multiple modes. For example, there are similar trends for segment II in the compaction phase of mode 9, for segment I in the compaction phase of mode 7, and both segments in the expansion phase of mode 8. Interestingly, when comparable ANM computations are applied to the structure of RNase A (PDB entry 7RSA) [28], all three types of breathing motion are detected (data not shown).

### Comparison with M23L-ONC·SO_4_

The structural details of superactive ONC mutants may offer valuable insight into the low ribonucleolytic activity of the wild-type enzyme. One such mutant, M23L-ONC, exhibits a five-fold elevation in RNA cleavage activity, a significant reduction in thermostability and an increase in susceptibility to proteolysis [37–39]. The C^α^ trace of M23L-ONC·SO_4_ as determined crystallographically at 100 K (PDB entry 1YV4) [9] bears a very strong resemblance to that of the present structure; the overall C^α^ deviation is 0.15 Å (rmsd), with the greatest deviation (0.37 Å) occuring at the mutation site. Using the rtONC·SO_4_ structure as a reference point, Merlino *et al.* [9] identified, in M23L-ONC·SO_4_, a difference in the conformation of Lys31 and a change in the hydrogen-bonding partner of its N^ζ^ atom (from Lys33 O in the wild-type to Asn34 O^δ1^ in the M23L mutant). They suggested that this placed it in a more favorable position to activate the enzymatic reaction [9]. The present structural analyses refine our view of these features: the disorder that we have observed in the Lys31 side-chain indicates that it is unlikely that a Lys31 N^ζ^···O Lys33 bond (or indeed any hydrogen bond involving Lys31 N^ζ^) exists in ONC·SO_4_ at room temperature or at 100 K. An important effect of the mutation, therefore, is the rigidification of the Lys31 side-chain. If viewed as a reduction in ground-state entropy, this represents one possible means by which catalysis is enhanced in this mutant. An additional means stems from the apparent overall increase in conformational flexibility (as indicated by the reductions in thermostability and resistance to proteolysis), which may favor the significant deformation necessary to attain the transition state.

Elsewhere across the substrate-binding site, there is little difference between the present structure and that of M23L-ONC·SO_4_. This extends to the disorder in the side-chain of Lys33.

## Discussion

**Figure 6:**
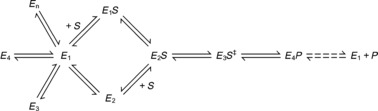
General scheme for the coupling of conformational change and catalysis in pancreatic ribonuclease superfamily members. *E*_1_ is the global conformational minimum and *E*_2_, *E*_3_ … *E*_n_ are local conformational minima along the catalytic pathway. After binding of the substrate (*S *) and attainment of the transition state (*E*_3_*S*^‡^), the reaction yields two products (*P *). In the case of a dinucleotide substrate (i.e. NpN′), the products are N 2′,3′-cyclic phosphate and N′. Release of the two products may involve multiple steps, as denoted by the dashed arrows.

It has become increasingly clear that the primary sequence of a protein encodes not only its equilibrium 3D structure, but also its intrinsic collective motion [40–42]. Multidisciplinary studies of model enzyme systems such as adenylate kinase have shown that these large-scale fluctuations in conformation can be coupled to catalysis [43]. It is likely that this is the case for RNase A, in which the rate constant for the predominant collective motion (1700 s^−1^) is the same as both *k*_cat_ and the rate constant for product dissociation [24–26]. Because patterns of backbone flexibility (in particular, the largest-scale collective modes) tend to be conserved within structurally homologous protein families [40–42], a proposed general conformational reaction scheme for catalysis by members of the pancreatic ribonuclease superfamily is presented in Fig. 6. Consistent with the behavior of RNase A, conformational minima (*E*_n_) that are intrinsic to the enzyme are mapped across the reaction sequence. In the absence of substrate, the enzyme samples a wide range of conformations (including those on the catalytic pathway), with the equilibria strongly favoring *E*_1_ (an open conformation that corresponds to the global energy minimum). The substrate is therefore, most likely to bind to *E*_1_ but, because the *E*_1_ → *E*_2_ conformational change is only a subtle closure, it could conceivably also bind to *E*_2_ or any of the conformational microstates inbetween. Substrate binding shifts the equilibrium to favor *E*_2_, from which the enzyme undergoes a further conformational change (to *E*_3_) to optimize complementarity to the transition state, most likely involving a more extreme closure [44]. Subsequent conformational changes effect release of the two products and complete the catalytic cycle. It follows that, when trying to understand the distinctive enzymatic properties of ONC in relation to those of RNase A, an attempt must be made to compare both the local active site structure and the relevant collective dynamics of each enzyme.

### Substrate-binding residues

A deficit in nucleotide-binding affinity accounts for a large proportion of the low ribonucleolytic activity of ONC relative to that of RNase A [13]. Previous crystallographic work on the binding of (deoxy)dinucleotides to the active site of each enzyme has indicated that enzyme–nucleotide interactions are not well optimized when these compounds initially bind to ONC [10,15]. At the P_1_ subsite, there is one less protein atom within hydrogen-bonding range of the phosphodiester linkage than at the same subsite of RNase A (in ONC: Lys9 N^ζ^, His10 N^ε2^ and Phe98 N; in RNase A: Gln11 N^ε2^, His12 N^ε2^, His119 N^δ1^ and Phe120 N), whereas the N^ζ^ atom of Lys31 is 1.3 Å further away from the nearest phosphoryl oxygen than is its counterpart, Lys41 N^ζ^, in RNase A (in ONC: 5.0 Å; in RNase A, 3.7 Å). Indeed, the side-chain of Lys31 in ONC does not contribute to ground-state nucleotide binding [13], unlike the side-chain of Lys41 in RNase A [45]. The findings of the present study imply that, despite the lack of a direct or water-mediated interaction between Lys31 and the nucleotide, the binding process brings about a rigidification of this residue. A comparable reduction in entropy is observed for the side-chain of Lys41 when nucleotide-free RNase A crystal structures (e.g. PDB entries 7RSA [28] and 1KF2 [22]) are compared with the RNase A·d(CpA) complex (PDB entry 1RPG [15]). By similar deduction, a rigidification of Lys33 at the B_1_ subsite of ONC also accompanies nucleotide binding. Although the equivalent residue in RNase A (Val43) shows the same tendency, its disorder is lower, being limited to discrete alternate conformations in the absence of nucleotides. Indeed, Val43 has substantially fewer degrees of freedom than does Lys33; thus, it is possible that an increased entropic penalty at this position contributes to the reduced nucleotide-binding affinity of ONC.

### Crystallographically-observed conformational variations

The reaction scheme in Fig. 6 demands that the enzyme possesses the requisite degree of large-scale conformational flexibility. In RNase A, this has been established experimentally via analysis of multiple crystal structures [23] and μs/ms-timescale solution dynamics [26]. In comparison, the number of available ONC crystal structures is small and no solution structure/dynamics of wild-type ONC have been reported. Our analyses of the present structure have, for the first time, identified appreciable collective motion in ONC. In all the analyses, the deduced motion has some similarity to that identified in RNase A in that it is greatest in the loops and helices that decorate the β-structure. Most of these loops are shorter in ONC than in RNase A (amounting to 20 fewer residues overall) and the locations of the longest loops appear to bias the motion toward one side of the protein (lobe 2). The magnitude of the deformation that occurs in response to cryocooling is modest but comparable to that measured for RNase A [19], whereas the effect of dinucleotide binding on these loops (in conjunction with crystallization in an alternative space group) is far more pronounced, and indeed more than when d(CpA) binds to RNase A [15].

The impact of the observed collective motion on the width of the active site cleft is of relevance to its potential role in catalysis. In the *E*_1_ conformation, the cleft is most likely significantly wider than that in RNase A [9]. To clarify how much wider, it would be helpful to compare the unliganded conformations of ONC and RNase A under closely matched conditions. Although suitable crystal and solution structures of RNase A are available [28,46,47], none of the ONC crystal structures reported to date (including the present structure) are unliganded and none of the ONC NMR structures are of the wild-type enzyme. In the absence of these data, a useful guide comes from comparison of the room-temperature, sulfate-liganded structures at pH 5.2–5.5. Using, as metrics, the Thr35 N···N Phe98 distance in ONC and the Thr45 N···N Phe120 distance in RNase A [8,9], the width of the cleft in ONC (9.17 Å; PDB entry 1ONC) [7] is ∼ 0.8 Å more than that in RNase A (8.36 Å; PDB entry 1KF2) [22]. It must be noted that even the binding of sulfate (a minimal substrate analogue) has a measurable effect on the global conformation of RNase A, reducing the cleft width of the unliganded enzyme by ∼ 0.2 Å (8.55 Å; PDB entry 7RSA) [28]. Unliganded ONC, therefore, may present a substrate-binding cleft with a width in excess of 9.17 Å.

The narrowing of the active site cleft caused by cooling ONC·SO_4_ crystals from room temperature to 100 K (0.31 Å) is comparable to that observed after cooling the P2_1_ crystal form of RNase A from 300 to 98 K (0.28 Å; PDB entries 8RAT and 1RAT, respectively) [19]. In addition, the marginal effect on cleft width observed on exchanging sulfate for d(UpGp) at the active site of ONC at constant temperature (a decrease of 0.10 Å) matches exactly that observed with sulfate- and d(CpA)-liganded RNase A (PDB entries 1KF2 and 1RPG, respectively) [15,22]. Hence, the possibility that the width of the active site cleft of ONC is modulated by an RNase A-like breathing motion remains open. A stronger stimulus of conformational change may allow a more extreme conformational of ONC to be trapped crystallographically or in solution. In the case of RNase A, the effects of the two environmental factors evaluated in the present work (temperature and dinucleotide binding) are surpassed by those resulting from the binding of pyrimidine nucleotide phosphates such as 2′-UMP [48] and 2′-CMP [8,15], which can be considered as product analogs. An even greater conformational change is likely to result from the binding of a transition state analog [44]. Indeed, a structural model for the ES^‡^ complex as it occurs in RNase A or ONC would constitute a major advancement in our understanding of the structural and dynamic requirments for catalysis by these enzymes. The crystallographically-observed binding mode of uridine vanadate, a marginal transition state analog [45], was not consistent with that of a transition state [49], so it is important that other transition state analogue candidates are evaluated.

Although one might hope to trap the ‘landmark’ conformations depicted in Fig. 6 via crystallization, it must be noted that each is surrounded by a large number of conformational microstates [50]. Because of the geometrical constraints of crystal packing, some of these may be much more conducive to lattice formation/preservation, leading to their prevalance over the landmark conformation. The influence of the lattice can be strong, perhaps most clearly illustrated by the *C*2 crystal form of RNase A in which one of the two protomers in the asymmetric unit (mol B) is constrained in a more open conformation than the other, irrespective of the ligand that is bound. For example, the active site cleft of mol B when bound to 2′-UMP is 0.65 Å wider than that of mol A in the same state, and is 0.53 Å wider than that of mol A in unliganded form (PDB entries 1O0M [47] and 2G8Q [48], respectively). Hence, it would be useful to study multiple crystal forms of ONC before drawing firm conclusions on its conformational flexibility.

### Intrinsic collective motion

In view of the present results and the tendency for the conservation of collective modes of backbone motion within structurally homologous protein families [40–42], it is most likely that the differences in sequence between ONC and RNase A have altered the relative populations of different conformational states and/or the kinetics of their interconversion [50]. NMR relaxation experiments on <Q1S-ONC suggest that the wild-type enzyme is conformationally more uniform than is RNase A [27], so the conformational landscape sampled by unliganded ONC may favor the open conformation (*E*_1_) to such an extent that the other conformational states are invisible to the technique. This need not be a large change from the equilibrium position measured for RNase A: it was calculated that a 3% upward shift in the relative population of *E*_1_ from its wild-type value of 95% would be sufficient to remove the NMR relaxation dispersion signal [51]. In addition, because the binding of a substrate or product analog to RNase A shifts the equilibrium to favor the respective closed conformation [26], NMR relaxation experiments conducted with ONC in the presence of such analogs may be more likely to reveal alternative conformational states [52].

Despite their simplicity, coarse-grained elastic network models such as the ANM have proven to be remarkably robust in their prediction of global protein dynamics [53]. The ANM analysis in the present study indicates that the largest-scale (lowest-frequency) collective modes of motion are conserved between RNase A and ONC. This includes three compaction/expansion variations, one of which resembles the familiar pincer-like breathing motion of RNase A. Although this breathing motion appears to predominate overwhelmingly in RNase A (as portrayed across numerous crystallographic ‘snapshots’ [23] and in the anisotropy of its high-resolution structure [54]), the findings of the present study suggest that this may not be the case for ONC. It is possible that, as a result of the more open *E*_1_ conformation of ONC, a more complex conformational change is required to effect catalysis. The ANM results are in contrast to the essential dynamics extracted from all-atom molecular dynamics simulations of 3–10 ns in duration, which highlighted a breathing motion in RNase A [23,36] but not in ONC [9]. Although atomistic molecular dynamics is considered to be the ‘gold standard’ for modeling protein fluctuations, the lowest-frequency modes of motion can be significantly under-represented if the simulation time is less than several hundred nanoseconds [55]. Therefore, it may be that longer molecular dynamics simulations are required to detect large-scale motion in ONC.

ONC now joins a select group of ribonucleases (comprising RNase A [22], RNase Sa [29] and eosinophil-derived neurotoxin [30]) for which an atomic resolution structure has been determined. The structure reported in the present study has enabled the re-interpretation of existing work and will serve as a valuable reference point for future structure–function studies on the protein.

### Note added in proof

After the submission of the present manuscript, the authors became aware of two unpublished ONC structures that have been deposited in the PDB. These are both low-temperature, sulfate-liganded structures in the same crystal form as the present structure (PDB entries 3HG6 and 3PHN at 1.70 and 1.46 Å resolutions, respectively). They exhibit modest conformational differences from the present structure, the nature of which are in accordance with the conclusions drawn above.

## Experimental procedures

### Crystallization and data collection

Authentic <Q1-ONC was purified from *R. pipiens* oocytes by a modification [56] of the original method [1]. Crystals were grown at 16 °C by the hanging-drop/vapor-diffusion method. In a refinement of previously established conditions [7], 2 μL of protein solution (10 mg·mL^−1^ in water) was mixed with an equal volume of reservoir solution [2.2 m (NH_4_)_2_SO_4_, 0.25% (w/v) poly(ethylene glycol) 4000, 0.01 m sodium acetate, pH 4.5], producing a rod-like crystal within 1 month. The crystal was flash-cooled to 100 K (using 25% glycerol) and diffraction data were collected on station 14.2 of the Synchrotron Radiation Source (Daresbury, UK). Images were processed using the hkl-2000 suite [57], as summarized in Table 1. The crystal was isomorphous with those grown under the parental conditions [7], containing one protein molecule per asymmetric unit.

### Structure determination and refinement

Structure factor amplitudes were calculated using the truncate procedure [58,59] and initial phases were obtained using the molecular replacement routines of amore [60], employing, as a search model, the ONC coordinates from PDB entry 1ONC [7]. All subsequent refinement adopted a maximum-likelihood target function and used a randomly-selected 5% of reflections for cross-validation [61]. Initial rounds of refinement were carried out with refmac5 [62] and were interspersed with electron density map viewing and manual model adjustments using coot [63]. During this time, isotropic ADPs were used. After inclusion of anions and water molecules, it was verified by sfcheck [64] that the diffraction data had no significant angular bias within the reciprocal lattice, anisotropic ADPs were adopted and then refinement proceeded with phenix [65]. The ADP X-ray weighting term *wxu_scale* was maintained at the default value of 1.0, whereas the stereochemical X-ray weighting term *wxc_scale* was increased from 0.5 to 1.5. For residues whose side-chains were observed in discrete alternate conformations, the occupancy of each conformation was refined (sum of occupancies = 1). The occupancies of three side-chains (Lys33, Lys78, Lys80) and three sulfate ions with single, weakly-defined conformations were also refined (occupancy < 1). In the final stages, hydrogen atoms were added in riding model positions. Structure validation was conducted with procheck [66], what_check [67], molprobity [68] and parvati [69]. Detailed refinement statistics are given in Table 2.

### Structural analysis

Electron density maps for previously published protein structures were obtained from the Uppsala Electron-Density Server [31]. Structural alignments were performed with vmd [70], as were mass-weighted radius of gyration calculations. Solvent-excluded volume was calculated using the Voronoi polyhedra approach [71] with a probe radius of 1.4 Å as implemented in vadar [72]. Before calculation of the solvent-excluded volume and radius of gyration, secondary side-chain conformations, hydrogen atoms, solutes and water molecules were removed from coordinate files. Difference distance matrix plots were generated using ddmp (Center for Structural Biology, Yale University, New Haven, CT, USA).

Anisotropic ADPs were analyzed using the prody package [73]. Briefly, for each C^α^ atom, the ADP matrix was diagonalized to obtain the three principal axes (eigenvectors) and their mean-square fluctuations (eigenvalues). At each position, the anisotropy, *A* was defined as the ratio of the smallest to the largest eigenvalue [69]. Intrinsic collective motion was investigated using the ANM [74] as implemented in prody. ANM calculations involved only C^α^ atoms and employed a uniform spring constant with a cut-off distance of 15 Å for interacting atoms. The six slowest nontrivial normal modes were examined. Molecular graphics were produced using vmd, nmwiz [73] and ccp4mg [75].

## Abbreviations

ADP: atomic displacement parameter
ANM: anisotropic network model
ONC: Onconase
PDB: Protein Data Bank
RNase A: ribouclease A
